# Nitric oxide‐mediated cutaneous microvascular function is not altered in middle‐aged‐to‐older adults following mild SARS‐CoV‐2 infection: A pilot study

**DOI:** 10.14814/phy2.15704

**Published:** 2023-06-03

**Authors:** Gabrielle A. Dillon, S. Tony Wolf, Auni C. Williams, W. Larry Kenney, Lacy M. Alexander

**Affiliations:** ^1^ Noll Laboratory, Department of Kinesiology The Pennsylvania State University, University Park State College Pennsylvania USA; ^2^ Center for Healthy Aging The Pennsylvania State University, University Park State College Pennsylvania USA; ^3^ Department of Anesthesiology and Perioperative Medicine Mayo Clinic Rochester Minnesota USA

## Abstract

We tested the hypothesis that post‐COVID‐19 adults (PC) would have impaired cutaneous nitric oxide (NO)‐mediated vasodilation compared to controls (CON). We performed a cross‐sectional study including 10 (10 F/0 M, 69 ± 7 years) CON and 7 (2 F/5 M, 66 ± 8 years) PC (223 ± 154 days post‐diagnosis). COVID‐19 symptoms severity (survey) was assessed (0–100 scale for 18 common symptoms). NO‐dependent cutaneous vasodilation was induced by a standardized 42°C local heating protocol and quantified via perfusion of 15 mM NG‐nitro‐L‐arginine methyl ester during the plateau of the heating response (intradermal microdialysis). Red blood cell flux was measured with laser‐Doppler flowmetry. Cutaneous vascular conductance (CVC = flux/mm Hg) was presented as a percentage of maximum (28 mM sodium nitroprusside +43°C). All data are means ± SD. The local heating plateau (CON: 71 ± 23% CVC_max_ vs. PC: 81 ± 16% CVC_max_, *p* = 0.77) and NO‐dependent vasodilation (CON: 56 ± 23% vs. PC: 60 ± 22%, *p* = 0.77) were not different between groups. In the PC group neither time since diagnosis nor peak symptom severity (46 ± 18 AU) correlated with NO‐dependent vasodilation (*r* < 0.01, *p* = 0.99 and *r* = 0.42, *p* = 0.35, respectively). In conclusion, middle‐aged and older adults who have had COVID‐19 did not have impaired NO‐dependent cutaneous vasodilation. Additionally, in this cohort of PC, neither time since diagnosis nor symptomology were related to microvascular function.

## 1 INTRODUCTION

The long‐term implications of SARS‐CoV‐2 infection are broad. Although these long‐term effects impact many organ systems, cardiovascular complications remain a primary concern. A wide range of cardiovascular complications have been reported with and without pathological evidence of cardiovascular disease. Twelve months post‐infection, adults who have been previously infected with SARS‐CoV‐2 still display greater risk for adverse cardiovascular events including venous thromboembolisms, angina, and heart failure (Raisi‐Estabragh et al., 2022; Xie et al., 2022). These cardiovascular incidents have been observed in both hospitalized and non‐hospitalized COVID‐19 patients (Raisi‐Estabragh et al., 2022; Xie et al., 2022). However, the pathophysiology linking COVID‐19 and increased cardiovascular risk remains elusive.

Preclinical data propose endothelial dysfunction as a long‐term consequence of COVID‐19 (Ackermann et al., 2020; Fosse et al., 2021; Qian et al., 2021; Varga et al., 2020). However, the literature in human participants is conflicting on whether COVID‐19 results in long‐term endothelial dysfunction in otherwise healthy adults (Dillon et al., 2022; Nandadeva et al., 2021; Ratchford et al., 2021). Endothelium‐dependent microvascular dysfunction is an early manifestation of the vascular disease process that precedes larger vessel dysfunction and clinically apparent target organ damage (Cohuet & Struijker‐Boudier, 2006; Dharmashankar & Widlansky, 2010; Khan et al., 2008). Specifically, impaired endothelial nitric oxide (NO)‐dependent vasodilation is evident in a variety of groups with elevated cardiovascular disease risk (Craighead et al., 2017; Cupisti et al., 2000; Greaney et al., 2020; Stanhewicz et al., 2017a; Stanhewicz et al., 2017b; Wolf et al., 2022). We have previously shown that young healthy adults (ages 19–35) do not display reduced NO‐mediated cutaneous microvascular function (Dillon et al., 2022). However, to‐date, no studies investigating NO‐mediated microvascular function in middle‐aged and older adults after recovery from COVID‐19 have been conducted. Considering aging in the absence of other cardiovascular disease risk is associated with reduced NO‐dependent vasodilation, and that older adults are more likely to experience complications following SARS‐CoV‐2 infection, we tested the hypothesis that compared to appropriately matched adults who have not had COVID‐19, middle‐aged and older adults who had been infected with SARS‐CoV‐2 would have impaired NO‐mediated cutaneous microvascular function (Seals et al., 2011; Taddei et al., 2001).

## 2 METHODS

The Institutional Review Board at The Pennsylvania State University approved all experimental procedures and protocols. Verbal and written informed consent were voluntarily obtained from all participants before participation and in accordance with the guidelines set forth by the Declaration of Helsinki.

### 2.1 Participants

All participants underwent a complete medical screening, including a blood chemistry analysis (Quest Diagnostics). All participants had a BMI ≤ 35 kg/m^2^ and did not use tobacco products. To increase generalizability to the public, we did not exclude for blood pressure status or any medications. All but one woman were postmenopausal. None of the postmenopausal women were on hormone replacement therapy. The one premenopausal woman (control group) was studied in the luteal phase of her menstrual cycle and urine pregnancy test confirmed the absence of pregnancy before the experimental procedures.

Participants were classified into one of two groups: (1) control (CON; never diagnosed with COVID‐19, no self‐reported COVID‐19 symptoms since January 2020) or (2) post‐COVID‐19 (PC; tested positive for SARS‐CoV‐2 via PCR test). In the PC group, COVID‐19 diagnoses were between January 2, 2021 and May 21, 2022. All data were collected between January 26, 2022 and July 14, 2022.

### 2.2 Symptom survey

At the experimental visit, PC participants completed a COVID‐19 symptomology questionnaire, which lists 18 symptoms commonly associated to COVID‐19, as previously described (Dillon et al., 2022; Ratchford et al., 2021; Stute et al., 2021). PC participants recalled the severity of their symptoms during peak COVID‐19 illness and for current symptoms at time of experimental testing.

### 2.3 Physical activity assessment

Regular exercise is associated with greater endothelial function, including upregulation of nitric oxide‐dependent pathways (Seals et al., 2009). Thus, we included a measure of habitual physical activity to ensure matching between groups. Daily physical activity was assessed using accelerometry (ActiGraph GT9X, LLC), as previously described (Dillon et al., 2022). Participants wore the accelerometer around their waist aligned with the midline of their dominant thigh (Freedson et al., 1998). A total of 15 out of 16 participants (CON *n* = 9 and PC *n* = 7) had sufficient data for analysis.

### 2.4 Cutaneous microvascular function

Before each experimental session, participants were instructed to abstain from caffeine, alcohol, and strenuous physical activity for at least 12 h before arrival at the laboratory. An intradermal microdialysis fiber (CMA Linear 31 probe, 55 kDa, Harvard Apparatus) was inserted into the ventral forearm skin for the local delivery of pharmacological agents, as previously described (Bruning et al., 2012; Dillon et al., 2022). Cutaneous red blood cell flux was continuously measured directly over the microdialysis site with an integrated laser Doppler flowmetry probe placed in a local heating unit (VP12 and VHP2; Moor Instruments).

Pharmacological agents were mixed just before use, dissolved in lactated Ringer's solution, sterilized using syringe microfilters (Acrodisc; Pall), and wrapped in foil to prevent degradation due to light exposure (FDA IND No. 120058). All solutions were perfused through the microdialysis fiber at a rate of 2 μL/min (Bee Hive controller and Baby Bee microinfusion pumps; Bioanalytical Systems).

After placement of the microdialysis fiber, ~60 min were allowed for hyperemia associated with fiber placement to resolve. Baseline data were then collected (∼10 min) before beginning a standardized local heating (42°C) protocol, as described previously (Choi et al., 2014; Minson et al., 1985). This local heating protocol elicits an initial axon reflex‐mediated peak skin blood flow response, followed by a brief nadir, after which there is a gradual rise and eventual blood flow plateau (after ∼40 min). After observing a stable local heating plateau, 15 mM NG‐nitro‐l‐arginine methyl ester (l‐NAME; NO Synthase Inhibitor) was perfused (Choi et al., 2014; Minson et al., 1985; Roberts et al., 2017; Roustit et al., 2010). After observing a stable l‐NAME plateau, 28 mM sodium nitroprusside (SNP; USP) was perfused and local temperature was increased to 43°C to elicit maximal vasodilation (National Institutes of Health, 2023; Tehrani & Gille‐Johnson, 2021). Automated brachial BP (Cardiocap; GE Healthcare; Connex Spot Monitor, Welch Allyn) was measured at each stage (e.g., baseline, initial axon reflex, local heating plateau, l‐NAME plateau, and maximal vasodilation) throughout the protocol.

### 2.5 Data and statistical analysis

Data were recorded at 40 Hz and stored for offline analysis (Powerlab/LabChart, ADInstruments). Average values for red cell flux (perfusion units) were obtained during baseline (averaged over 5–10 min) and at each phase of the local heating protocol (averaged over ≥1 min). Cutaneous vascular conductance (CVC) was calculated as red blood cell flux divided by mean arterial pressure. Because of the heterogeneity of capillary density at each microdialysis site, CVC was normalized as a percentage of the site‐specific maximum (CVC%max) (Roberts et al., 2017; Roustit et al., 2010). The NO‐dependent vasodilation (%NO) was quantified as the difference between the local heating plateau and the l‐NAME plateau (Choi et al., 2014; Roberts et al., 2017; Roustit et al., 2010; Wolf et al., 2020).

Participant characteristics were analyzed using an unpaired *t* test (IBM Corp. Released 2019. IBM SPSS Statistics for Windows, v. 26.0.; GraphPad Prism v. 9.0.0 for Windows, GraphPad Software). Responses to local heating were analyzed using a two‐way anova to evaluate group (CON and PC) and phase (baseline, plateau, l‐NAME, and maximum) effects. The NO contributions were analyzed using an unpaired *t* test. Linear regression analyses were performed using a Pearson's correlation coefficient to assess the associations between (1) time since COVID‐19 diagnosis and the NO contribution to the cutaneous vasodilation response during local heating and (2) COVID‐19 symptomology and the NO contribution to the local heating response. Significance was set a priori at *α* < 0.05. All results are presented as means ± SD, and range is displayed as minimum to maximum.

## 3 RESULTS

### 3.1 Participants

A total of 17 adults participated in the pilot study: 10 CON (10 F/0 M) and 7 PC (2 F/5 M). Height and weight were greater in the CON compared to the PC group (Table 1). All other participant characteristics, including body mass index, were not different between groups (all *p* > 0.05, Table 1). Overall physical activity and time spent in each activity domain (i.e., sedentary, light, and moderate‐to‐very vigorous) was not different between groups (all *p* > 0.05, Table 1).

**TABLE 1:** Participant characteristics.

	Control (*n* = 10)	Post‐COVID‐19 (*n* = 7)
Age (years)	69 ± 7, 54–80	66 ± 8, 52–75
Height (cm)	163 ± 3, 158–166	175 ± 9, 160–186*
Weight (kg)	71 ± 11, 53 95	86 ± 16, 64–114*
Body mass index (kg/m^2^)	27 ± 4, 20–35	28 ± 4, 21–35
Systolic blood pressure	123 ± 7, 108–130	127 ± 15, 103–150
Diastolic blood pressure	76 ± 5, 67–83	76 ± 10, 61–90
Stage I hypertension	*n* = 1 (10%)	*n* = 1 (14%)
Stage II hypertension	*n* = 2 (20%)	*n* = 1 (14%)
Heart rate (bpm)	66 ± 14, 48–90	66 ± 9, 58–84
HDL‐C (mg/dL)	65 ± 13, 45–84	57 ± 13, 41–77
LDL‐C (mg/dL)	130 ± 1,4107–160	113 ± 29, 71–158
Dyslipidemia	*n* = 3 (30%)	*n* = 2 (29%)
HbA1c (%)	5.4 ± 0.2, 51.‐5.7	5.5 ± 0.3, 4.9–5.9
Medications		
Aspirin	*n* = 3 (30%)	
Antihypertensive	*n* = 4 (40%)	*n* = 2 (29%)
Statin	*n* = 3 (30%)	*n* = 1(14%)
Physical activity (min/day)	*n* = 9	*n* = 7
Sedentary	534 ± 107 (319–656)	561 ± 63 (462–655)
Light	271 ± 90 (101–423)	268 ± 90 (165–348)
Moderate‐to‐very vigorous	26 ± 12 (6–46)	19 ± 15 (6–42)
Steps (steps/day)	6964 ± 2253 (1990–9963)	6487 ± 2161 (3823–8838)

Abbreviations: HDL‐C, high‐density lipoprotein cholesterol; LDL‐C, low‐density lipoprotein cholesterol.

* *p* < 0.05 versus control.

Within the CON group, *n* = 8 received three doses of the COVID‐19 vaccine (Pfizer: *n* = 3 and Moderna: *n* = 5), *n* = 1 received two doses of Moderna, and *n* = 1 was not vaccinated for COVID‐19. Within the PC group, *n* = 4 received three doses of the COVID‐19 vaccine (Pfizer: *n* = 2 and Moderna: *n* = 2), 1 received one dose of Pfizer, and 2 were not vaccinated for COVID‐19.

### 3.2 Blood flow responses

**FIGURE 1:**
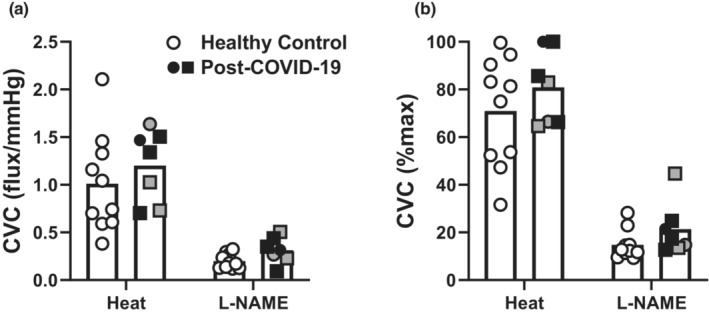
The absolute (panel A) and relative (panel B) cutaneous vascular conductance (CVC, panel A) responses during the heating and l‐NAME plateaus of a 42°C local heating protocol were not different between controls (*n* = 10, open symbols) and post‐COVID‐19 adults (*n* = 7, closed symbols). The gray symbols indicate the three individuals who reported post acute sequelae COVID‐19 symptoms at the time of testing. Females are represented as circles and males are squares. Two‐way anova results: Panel A: Phase *p* < 0.01, Group *p* = 0.25, Interaction *p* = 0.74; Panel B: Phase *p* < 0.01, Group *p* = 0.14, Interaction *p* = 0.77.

**FIGURE 2:**
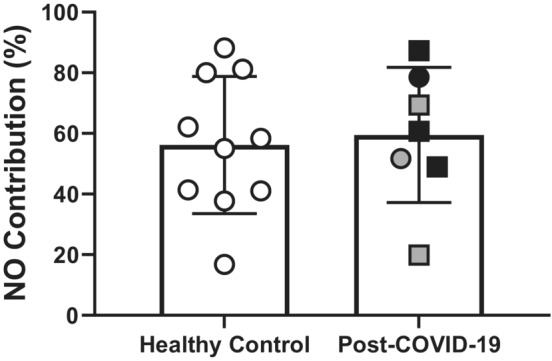
The nitric oxide (NO) contribution to the 42°c local heating protocol was not different between controls (*n* = 10) and post‐COVID‐19 adults (*n* = 7). The gray symbols indicate the three individuals who reported post acute sequelae COVID‐19 symptoms at the time of testing. Females are represented as circles and males are squares. Unpaired *t* test: *p* = 0.77.

Baseline, initial axon reflex‐mediated peak, and maximum CVC values, whether expressed as absolute (flux/mm Hg) or relative (CVC%max) did not differ between groups (Table 2). The local heating plateau and L‐NAME plateau values are presented in Figure 1. There were no differences between groups at either phase, regardless of whether expressed as absolute or relative CVC. Similarly, the NO contribution to the local heating response did not differ between groups (Figure 2).

**TABLE 2:** Cutaneous blood flow responses to local heating.

	Control (*n* = 10)	Post‐COVID‐19 (*n* = 7)	*p*‐values
Absolute CVC (flux/mm Hg)			
Baseline	0.2 ± 0.1 (0.1–0.3)	0.1 ± 0.0 (0.1–0.3)	Phase *p* < 0.01, group *p* = 0.785, interaction *p* = 0.921
Axon reflex	0.7 ± 0.4 (0.4–1.6)	0.7 ± 0.5 (0.4–1.4)	
Maximal vasodilation	1.4 ± 0.6 (0.8–2.8)	1.5 ± 0.5 (0.7–2.0)	
Relative CVC (%CVCmax)			
Baseline	10.4 ± 2.6 (6.6–14.6)	9.2 ± 4.8 (3.4–16.3)	Phase *p* < 0.01, group *p* = 0.979, interaction *p* = 0.785
Axon reflex	47.1 ± 14.3 (30.6–69.9)	48.1 ± 21.2 (25.6–69.9)	

Abbreviation: CVC, cutaneous vascular conductance.

### 3.3 Timeline and symptomology

The time since COVID‐19 diagnosis (223 ± 154 days, range: 46–430 days) was not related to the magnitude of NO contribution (*r* < 0.01, *p* = 0.99). All PC adults experienced symptoms during COVID‐19 illness (Table 3). The number of COVID‐19 symptoms (9 ± 3 symptoms, range: 3–12) and the average symptom severity (46 ± 18 AU, range: 27–79) were not related to the magnitude of the NO contribution (number of symptoms: *r* = 0.58, *p* = 0.17; symptom severity: *r* = 0.42, *p* = 0.35). Three of the seven PC participants (1 F/2 M) reported experiencing COVID‐19‐related symptoms at the time of testing (number of symptoms: 5, 3, and 2 symptoms; symptom severity: 6, 20, and 10 AU; days since diagnosis: 430, 220, and 162 days, respectively).

**TABLE 3:** Peak symptom severity recall in post‐COVID‐19 adults.

Post‐COVID‐19 participant	1	2	3	4	5	6	7
Chest pain	95	10		40			
Chills				50		40	30
Diarrhea	5			100		20	
Dizziness	80						
Dry cough	100	80	50	100		30	10
Dry eyes							
Dry mouth							
Fatigue	85	100	10	100	80	50	40
Fever (over 100.3 F)	5	5		100		50	30
Headache	5	20		20	40	20	30
Lack of appetite	5	10			50	20	20
Loss of smell/taste							
Muscle or body aches	15	35		90	40	30	30
Nasal congestion or runny nose	90	50	50	80		10	30
Nausea or vomiting				100			
Shortness of breath	100	70			60		
Sore joints		45		90	40	10	30
Sore throat	100					20	30
Total number of Sx	12	10	3	11	6	11	10
Average severity/Sx	57	43	37	79	52	27	28

## 4 DISCUSSION

The NO contribution to the cutaneous vasodilation response during local heating was not different between middle‐aged and older adults who have had COVID‐19 and those who have not. Additionally, neither the time since COVID‐19 diagnosis nor symptom severity were correlated with the NO contribution to the local heating response in middle‐aged and older adults who have had COVID‐19.

Similar to our previous data in young adults (Dillon et al., 2022), the present data suggest that there are no differences in NO‐mediated cutaneous microvascular function in middle‐aged and older adults who have had mild COVID‐19 and recovered compared with those who have not. In general, these data suggest mild COVID‐19 illness is not associated with NO‐mediated microvascular dysfunction in middle‐aged and older adults. Whether NO‐mediated endothelium‐dependent microvascular dysfunction is an underlying mechanism for post acute sequelae COVID‐19 (PASC)‐cardiovascular syndrome or PASC‐cardiovascular disease is still unknown. Although three of the seven PC participants had PASC, this study was not designed nor adequately powered to assess potential differences in microvascular function in adults with PASC relative to those without PASC.

All PC participants experienced mild COVID‐19 symptoms and none were hospitalized due to SARS‐CoV‐2 infection (National Institutes of Health, 2023). Thus, the current findings are limited to middle‐aged and older adults who have experienced only mild COVID‐19 symptoms. Impaired endothelium‐dependent cutaneous vasodilation has been observed in middle‐age and older adults who were hospitalized due to COVID‐19 (Raia et al., 2022; Tehrani & Gille‐Johnson, 2021). However, the mechanism(s) underlying that impairment (e.g., reduced NO bioavailability) are unknown. A participant cohort that includes PC patients who experienced moderate‐to‐severe COVID‐19 symptomology (e.g., hospitalization, respiratory failure) may be needed to observe a significant relation between COVID‐19 symptomology and microvascular function. Furthermore, although we did not observe any individual relations between microvascular function and post‐recovery timing or symptomology, there may be a combined impact of timeline and symptomology, as well as other factors such as preexisting comorbidities, fitness, and vaccine status. A comprehensive phenotyping of these factors and their relation with microvascular function in PC individuals is beyond the scope of the present study, warranting future investigations to better understand the underlying factors that result in disparate impacts of COVID‐19 on microvascular endothelial function.

There are several limitations to the present pilot study. First, this small sample was a random sample of individuals who had varying times of infection, and thus were likely infected by different SARS‐CoV‐2 variants, and a wide range of symptomology, including those with both PASC and those who had fully recovered. A recent study has suggested differential impacts of COVID‐19 variants on human microvascular function in isolated human arterioles (Nishijima et al., 2023). In the present study, testing was not performed to determine the COVID‐19 variants with which the participants were infected, so we are unable to determine whether there are differential impacts of COVID‐19 variants on microvascular function in vivo. Future in vivo investigations should include samples that are appropriately powered to delineate the impact of COVID‐19 variants and other individual determinants of illness severity and PASC. The goal of this study was to determine if, in general, SARS‐CoV‐2 infection was associated with microvascular dysfunction in middle‐aged and older adults. Second, despite an effort to enroll an equal number of sexes between groups, the groups were unmatched for sex. All of the controls were female, while only two out of the seven PC adults were female (*n* = 5 were male). Sex differences in endothelial vascular function throughout the lifespan are still being elucidated. In general, males experience a decline in endothelial function earlier in life compared to females (Green et al., 2016; Stanhewicz et al., 2018). Thus, females typically have better endothelial function compared to males until 50–60 years of age, after which endothelial function declines more rapidly in females compared to males (Green et al., 2016; Merz & Cheng, 2016; Stanhewicz et al., 2018). Furthermore, sex differences in the infection rate, clinical presentation of COVID‐19, and COVID‐19 long‐term impacts have been observed. Many, but not all, investigations report COVID‐19‐related morbidity and mortality rates are greater in males compared to females (Danielsen et al., 2022; Pelà et al., 2022; Scully et al., 2020; Yao et al., 2020). Mechanisms underlying these sex differences have not been identified. In the present study we did not observe any obvious sex differences. However, future research is warranted regarding the potential interactions between sex, vascular function, and the long‐term implications of COVID‐19.

In conclusion, these pilot data suggest middle‐aged and older adults who have recovered from mild COVID‐19 do not display impaired NO‐mediated cutaneous microvascular function. Time since COVID‐19 diagnosis and COVID‐19 symptomology were not related to microvascular function.

## CONFLICT OF INTEREST STATEMENT

No conflicts of interest, financial or otherwise, are declared by the authors.
